# Proof of Concept of Natural and Synthetic Antifouling Agents in Coatings

**DOI:** 10.3390/md22070291

**Published:** 2024-06-24

**Authors:** Daniela Pereira, Joana R. Almeida, Honorina Cidade, Marta Correia-da-Silva

**Affiliations:** 1 Laboratory of Organic and Pharmaceutical Chemistry, Department of Chemical Sciences, Faculty of Pharmacy, University of Porto, 4050-313 Porto, Portugal; up2009004461@edu.ff.up.pt; 2 Interdisciplinary Centre of Marine and Environmental Research (CIIMAR), University of Porto, 4450-208 Matosinhos, Portugal; jalmeida@ciimar.up.pt; 3 UNIPRO—Oral Pathology and Rehabilitation Research Unit, University Institute of Health Sciences (CESPU), 4585-116 Gandra, Portugal

**Keywords:** marine biofouling, natural products, synthesis, antifouling, antibiofilm, coatings, in situ validation

## Abstract

Marine biofouling, caused by the deposition and accumulation of marine organisms on submerged surfaces, represents a huge concern for the maritime industries and also contributes to environmental pollution and health concerns. The most effective way to prevent this phenomenon is the use of biocide-based coatings which have proven to cause serious damage to marine ecosystems. Several research groups have focused on the search for new environmentally friendly antifoulants, including marine and terrestrial natural products and synthetic analogues. Some of these compounds have been incorporated into marine coatings and display interesting antifouling activities caused by the interference with the biofilm-forming species as well as by the inhibition of the settlement of macroorganisms. This review highlights the proof-of-concept studies of emerging natural or synthetic antifouling compounds in coatings, from lab-made to commercial ones, performed between 2019 and 2023 and their results in the field or in in vivo laboratorial tests.

## 1. Introduction

The phenomenon of marine biofouling is responsible for several negative economic, environmental, and safety-related issues. The accumulation of marine micro- and macroorganisms in submerged surfaces increases surface roughness and irregularities, thereby increasing the frictional resistance of the ships through water, consequently increasing fuel consumption and the emission of greenhouse gases [1,2,3]. This process also contributes to the corrosion of equipment and to an increase in the frequency of the dry-dock period for additional hull cleanings. The settlement of fouling organisms on hull vessels is also a major issue concerning the introduction of invasive, nonindigenous species into non-native environments, being a potential hazard to the local marine ecosystems. The impact of this phenomenon can be ecological and evolutionary, with direct and indirect competition with native species, effects on higher trophic levels, and changes in ecosystem processes, as well as economic and societal, including management costs, impacts on human health, and costs for eradication and control measures [4,5,6,7].

Biocide-based antifouling (AF) coatings have become the most common way to prevent biofouling due to their efficiency, low cost, and facility of maintenance. Copper and other heavy metals (arsenic, mercury) have been used as biocides for several centuries. In the 1960s, tributyltin (TBT) was introduced as a biocide in marine AF coatings and became the dominant AF agent. However, due to the high toxicity of organotins to several aquatic organisms, TBT was banned by the International Maritime Organization (IMO) in 2008. Subsequently, copper and zinc have been gradually used to replace organotins. Booster biocides, such as cybutryne (Irgarol 1051^®^), zinc pyrithione, dichlofluanid, chlorothalonil, and 4,5-dichloro-2-n-octyl-4-isothiazolin-3-one (Sea Nine 211^®^), among others, have also been applied to AF coatings. However, several studies have shown that these AF alternatives can also bioaccumulate in marine organisms and cause serious damage to marine ecosystems [6,8,9,10,11]. At the 76th session of the Maritime Environmental Protection Committee, the use of cybutryne in antifouling systems was prohibited by the IMO from 1 January 2023, since studies have demonstrated that this substance is harmful to several marine organisms.

Even though the commercially available biocides can present some hazards to the environment, coatings containing biocides are the most widely used [7].

To face the potential risks of currently used biocides, several studies have reported the potential of natural compounds extracted from marine and terrestrial sources, with interesting AF properties [12,13,14,15,16,17]. In addition to them, some nature-inspired synthetic AF derivatives have also been developed and shown to display eco-friendly features and low ecotoxicity [18,19,20,21,22].

Regarding coating principals, different strategies have been applied to pursue AF efficacy, including bioinspired approaches (biomimetic microstructures/textures, natural bioactive additives, and bioinspired polymer matrices like hydrogels, slipper liquid-infused porous surface (SLIPS), zwitterionic polymers, and other biodegradable matrices) [5,7,23,24,25,26]. The principle of biodegradable coatings lies in the fact that a surface constructed from a biodegradable polymer would gradually decompose and erode under seawater or through enzymatic action in a marine environment, which can polish the attached living organisms or inorganics, leading to a self-renewal surface. Various research groups have developed coatings based on biodegradable polyurethanes with interesting AF potential and without a negative impact for the marine environment [27,28,29].

Among the chemically active AF technologies, which act through the controlled release of biocides, two main categories have arisen: insoluble matrix—contact leaching coatings—and soluble matrix—controlled depletion coatings (CDPs) and self-polishing coatings (SPC) [24]. Due to their short lifetimes, insoluble matrix coatings are currently less commonly used. Soluble matrices are less expensive compared with insoluble matrix coatings and have a good roughness control and therefore they are preferred. 

Those categories differ either in the nature of the matrix (epoxy, rosin, acrylic, silicone, polyurethane, among others) and/or the behavior of the biocides release. 

Although the polymeric matrices are the essence of the coatings, other constituents, such as solvents or diluents, additives, pigments, crosslinkers, and extenders, are also essential for the coating formulation [30]. 

This review compiles the reported studies concerning the incorporation of small molecules obtained from natural resources or through chemical synthesis in coatings, and assessments of their real AF efficacy in situ using laboratorial or field validation tests. Herein, we report the results published in the last five years (2019–2023), divided according to the type of coating used and presented in each section in chronological order. The chemical structures of the studied compounds, the nature of the polymers, and the other constituents essential for the coating formulation are highlighted.

## 2. Laboratory-Prepared Coatings Containing Natural and Synthetic Antifoulants

### 2.1. Acrylic Resin-Based Coatings

Acrylic- or methacrylic-based coating is decomposed upon contact with seawater by the hydrolysis of the pendant groups of the acrylic or methacrylic copolymer chain, leaving a fouling-free surface [4]. These coatings are classified as SPC. SPC coatings started to be applied in the 1970s and serve as an important advancement in the development of AF materials. The acrylic copolymer is mixed with the biocide that gives the SPC a smooth surface and the biocide leaching rate is controlled by the rate of resin erosion [4,24]. The typical polishing rate of the coating is approximately 5–20 µm per year and the service life can be up to 5 years [4,31,32].

**Figure 1:**
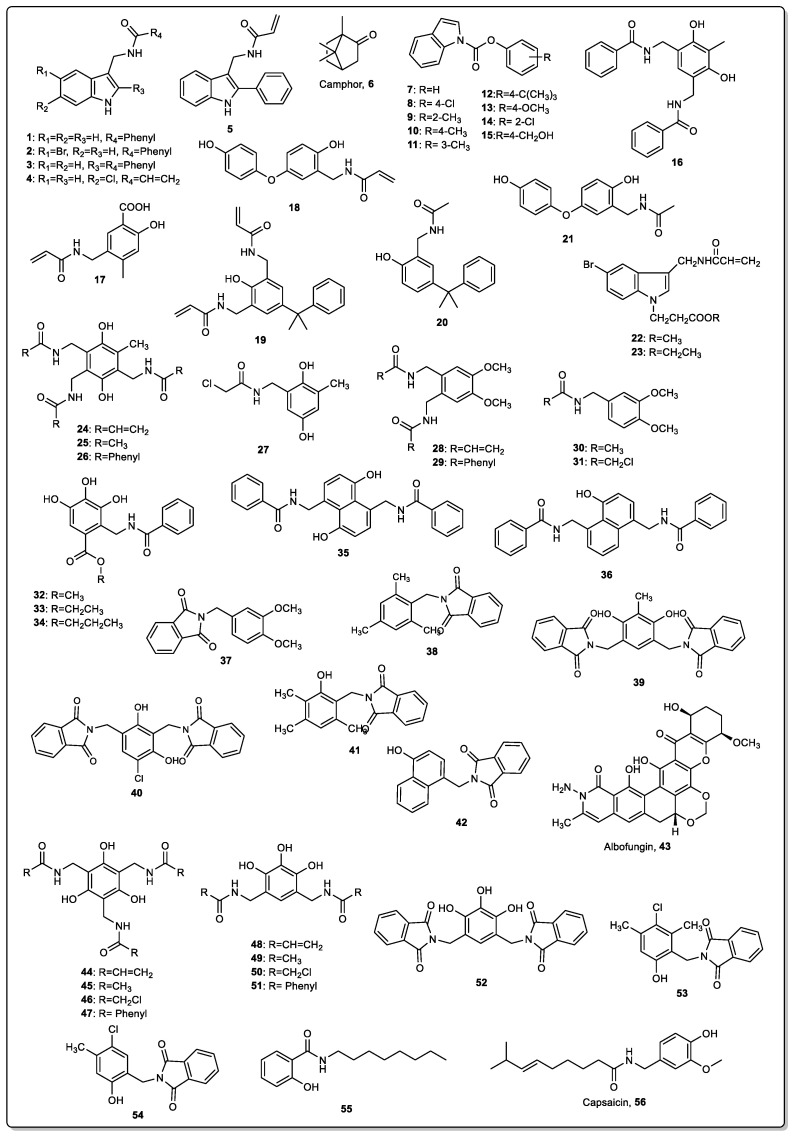
Structures of AF compounds **1**–**56**.

Some indole derivatives obtained from marine organisms have shown potential AF activity against macro- and microfouling species [33,34]. Based on these indole natural products with AF activity, seven indole derivatives were synthesized by Feng et al. and evaluated for their effectiveness in preventing biofouling [35]. All compounds displayed activity against diatoms *Phaeodactylum tricornutum Bohlin*, *Nitzschia closterium*, and *Platymonas subcordiformis* and antibacterial activity against bacteria *Staphylococcus aureus* and *Escherichia coli*. These compounds were included at 5% in a coating composed of resins (23%), Cu_2_O (25%), pigment Fe_2_O_3_ (36%), xylene (8.5%), and an additive (2.5%). The commercial biocide copper pyrithione was used as a reference for AF property. The prepared coatings were brushed on treated PVC panels and were immersed in seawater at one meter deep for five months. Although abundant algae and biofilms emerged on the control panel without bioactive compounds, only a few biofilms appeared on the panels containing coatings with compounds **1**–**4** (Figure 1). Overall, coatings containing compounds **2** and **3** displayed better AF performance compared with the coating with reference antifoulant [35]. The same research group also reported the synthesis of another indole derivative (**5**) and its evaluation as an antifoulant [36]. Compound **5** presented antibacterial activity against *E. coli* and *S. aureus*, as well as prominent inhibitory activity against marine diatoms *Phaeodactylum tricornutum*, *Nitzschia closterium*, and *Skeletonema costatum* and was further incorporated into acrylate resins at 0%, 5%, 10%, and 15%, which were submitted to algal inhibition and AF tests. The resins were composed of monomers, butyl acrylate (4 molar ratio), ethyl acrylate (4 molar ratio), acrylic acid (1 molar ratio), methacrylic acid (1 molar ratio), and Zn(OH)_2_ (2 molar ratio), and solvents, xylene and n-butyl alcohol. The self-polishing performance of the acrylate resins, evaluated by the abrasion quantity during the dynamic simulation and the static immersion experiments, showed that the acrylate resins to which the indole derivative was introduced still exhibited prominent polishing properties. Acrylate resins containing compound **5** showed higher algal inhibitory activity against *Nitzschia closterium* and *Phaeodactylum tricornutum* than acrylate resins without the bioactive compound, with the algal inhibitory activity being greater with increases in the amount of compound **5**. The prepared resins were also coated on PVC panels and immersed in the sea at one meter deep for three months. After this time, panels with indole derivative were much less covered with fouler organisms than panels with only the resin mixture, particularly the panels with the highest amount of indole derivative incorporated into acrylate resins (10% and 15%) [36].

Hu et al. reported the development of a new polymer AF coating using environmentally friendly terpolymer resins and the natural AF agent camphor (**6**, Figure 1) [37]. The acrylic resins were composed of triisopropylsilylacrylate (TIPSA), isobornyl methacrylate (IBOMA), n-butyl methacrylate (BMA), camphor, and xylene. Six different resins with variable proportions of polymers were synthesized. Camphor was added at a 10% camphor:resin ratio. Researchers also added 10% rosin to one of the prepared acrylic resins in order to study the effect of rosin on the regulation of the release rate of camphor. Studies to evaluate the performance of the release of antifoulant camphor from the paint were performed and showed that the changes in TIPSA content had no significant effect on the release rate and AF performance of the resin system. The change in IBOMA content did not significantly affect the release rate of the camphor, although the decrease in IBOMA content decreased the AF performance of the resin system. They also showed that the addition of rosin to the acrylic resin system could help to regulate the release rate of the resin system by reducing the release rate of camphor in the early stage and accelerating the release rate of camphor in the later stage. The previously prepared coatings were applied to steel panels (300 × 250 × 3 mm) and immersed in the sea with a depth interval of 1.4–1.75 m for nine weeks. After this time, all the coatings presented a lower number of barnacles attached than on the control coating. It was found that for coatings with a lower amount of IBOMA, the AF performance of the resin system slightly decreased, which can be explained by the decreased release of borneol, a constituent of the polymer that contains antimicrobial properties [37].

Inspired by the promising AF activity of natural indoles, a series of indole derivatives (compounds **7**–**15**, Figure 1) were prepared by introducing a carboxylic acid aryl ester at the *N*-position of the indole ring. All indole-1-carboxylic acid aryl esters displayed AF activity and were submitted to a field test in a resin-based paint [38]. Paints were prepared by adding 90 wt.% of acrylic ester resin with 10 wt.% of compounds **7**–**15**, and then were coated onto the surface of the steel panels (300 × 150 × 3 mm). Control paints without any compound or with indole were also prepared. After the immersion of the panels in seawater at a depth of 0.5–1.0 m for three months, it was found that coatings containing the synthesized compounds were shown to be more effective in the inhibition of the adherence of barnacle larvae on sheet steel than the indole and blank coatings, which were highly fouled by marine organisms, including barnacles, oysters, and other fouling organisms. Moreover, it was found that the presence of electron-withdrawing groups was more beneficial for AF activity than electron-donating groups. The evaluation of the release rates of compounds from the AF coatings showed a downward trend as time prolonged, at first having a higher release rate due to the massive release from the surface layer, which stabilized after 14 days, resulting in the long-lasting and effective inhibition of the growth and attachment of marine fouling organisms [38].

Six synthetic analogues of capsaicin, a natural compound with AF activity, (compounds **16**–**21**, Figure 1) were synthesized by Wang et al. and their AF effect against three species of diatoms (*Phaeodactylum tricornutum*, *Skeletonema costatum*, and *Chaetoceros curvisetus*) was studied [39]. These compounds were shown to be active against these microfouling organisms and were submitted to a field test by incorporating the obtained compounds at 10% into a coating composed of an acrylic resin (55 g), copper oxide (25 g), iron oxide (17 g) as a pigment, bentonite, and polyamide wax (2 g) as auxiliaries, and xylene (11 g) as a solvent. A control paint without active compounds was also prepared. The AF coatings were applied to steel panels (300 × 150 × 3 mm^3^) and immersed in seawater for three months at a depth of 0.2–2 m. After the exposure time, compounds-containing coatings were less fouled by microorganisms and barnacles than the control [39].

Later, based on the AF potential of the indole compounds found in a marine environment, Ni et al. reported the field assay of two indole ester derivatives containing the acrylamide group (compounds **22** and **23**, Figure 1) after incorporation at 10% into a marine coating composed of resin (30%), copper oxide (I) (20%), pigments, fillers (25%), and solvent (15%) [40]. A coating with the commercial biocide chlorothalonil, used as a positive control, was also prepared. After immersion of the coated steel plates (250 mm × 150 mm × 3 mm) at 1–1.5 m for 24 months, the blank plate was fully covered with macrofouling organisms, such as mussels, while coatings containing the two compounds were only colonized by a soft biofilm coverage. Moreover, the anti-protein adsorption experiment of AF paints with indole derivative and chlorothalonil as AF agents proves that indole derivatives exhibit better protein resistance behaviors than the positive control, which is propitious to inhibit the formation of fouling in marine environments, particularly at the early stage. Overall, these coatings were found to present better AF performance than coatings prepared with reference antifoulant chlorothalonil [40].

In another study, eight capsaicin derivatives (**24**–**31**, Figure 1) were synthesized by Wang et al. and studied for their effect against marine biofouling [41]. Further, these compounds were incorporated at 4.8% into a coating prepared with zinc acrylic resin (28.2%), Cu_2_O (34%), Fe_2_O_3_ (5.1%), ZnO (5.9%), talcum powder (6.4%), bentonite (0.2%), rosin resin (9.4%), auxiliaries (4%), and xylenes (2%). PVC panels were coated with previously prepared paints and immersed in seawater at a depth of 0.2–2 m for six months. A panel coated with a control paint without capsaicin derivatives was also tested. Compared with the control paint that only contained cuprous oxide as an antifoulant, panels (300 mm × 150 mm × 3 mm) coated with paints containing capsaicin derivatives as adjuvant antifoulants were less fouled, showing that the AF potential of the coatings was enhanced by the addition of capsaicin derivatives [41].

Another five capsaicin derivatives (**32**–**36**) were also reported by Wang et al. for their potential as AF adjuvants [42]. At a concentration of 4.8%, these compounds were incorporated into a coating as previously described [41] and used to coat PVC panels. A coating containing biocide chlorothalonil was used as a positive control and a coating containing capsaicin derivatives was used as test samples. After the immersion of panels for six months, the presence of biofilms and barnacle larvae was found in the control panel, whereas less biofilm coverage and barnacle larvae presence were found in the test plate, which confirmed the AF activity, in particular for compounds **32**–**34** [42].

An et al. reported the synthesis and AF evaluation against marine bacteria and algae of six new derivatives of the natural compound capsaicin containing amide groups (compounds **37**–**42**, Figure 1) [43]. Compounds **40**–**42** presented the highest activity, but all the compounds were incorporated into a marine coating and submitted to a field test. The coating was composed of acrylic resin (55 g), pigment (17 g), auxiliary materials (2 g), xylene (12 g), and capsaicin derivative (25 g). A control paint composed of acrylic resin (55 g), pigment (17 g), auxiliary materials (2 g), xylene (12 g), and copper oxide (25 g) was also prepared for comparative purposes. PVC panels were coated and immersed in seawater at a 2 m depth for three months. Compared with the control panel without AF coating or a panel containing AF coating without compounds **37**–**42**, all coatings presenting capsaicin derivatives presented lower amounts of fouling marine organisms, with coatings with compounds **40**–**42** being the most promising in this assay [43].

She et al. found that the natural product albofungin (**43**, Figure 1) and some derivatives, isolated from the metabolites of bacterium *Streptomyces chrestomyceticus* BCC 24770, had potent antibiofilm activities against Gram-positive (*Micrococcus* sp. Z02, *S. aureus* B04 and *Staphylococcus* sp. Z01) and Gram-negative (MCCC 1A04899 *Sulfitobacter pontiacus*, MCCC 1A01390 *Pseudomonas pachastrellae*, and MCCC 1A11723 *Psychrobacter nivimaris*) marine bacteria and anti-macrofouling activities against the larval settlement of fouling organisms with low cytotoxicity [44]. Considering that albofungin (**43**) showed the best AF activity and the highest yield in the bacterial culture, this compound was incorporated into a coating based on methyl methacrylate (MMA) and tributylsilyl methacrylate (TBSM) copolymer (PMSM0), which was synthesized via radical ring-opening polymerization, and the AF compound was incorporated in concentrations of 5, 10, and 15%. For 5 wt.% of albofungin-based coating, PMSM0 (0.95 g) and albofungin (**43**) (0.05 g) were dissolved in xylene and tetrahydrofuran (v:v, 1:2) and mixed vigorously at room temperature. PVC panels (4 × 7 cm^2^) were coated with the previously prepared solutions and, after being dried at room temperature for 7 days, were submersed in the sea for two months at a depth of 0.5 m. After this time, panels containing compound **43** in the three concentrations tested (5, 10, and 15%) were much less fouled than the control paint, which was 96% covered by macrofoulers. The release rate of albofungin from coatings at different concentrations into artificial seawater was measured within 35 days. During the course of the observation period, the release rate was generally low and positively correlated with a concentration of albofungin [44]. 

Inspired by the antimicrobial potential of phloroglucinol, a series of polyphenol derivatives was synthesized by Wang et al. and tested for AF activity in vitro and after incorporation into an acrylic resin-based coating [45]. Compounds **44**–**51** (Figure 1) displayed antibacterial activity against *S. aureus*, *E. coli*, and *P. aeruginosa* and antialgal activity against microalgae *Chlorella vulgaris* and *Nitzschia Closterium*. Compounds **44**–**51** (4.8 g) were then incorporated into a coating composed of an acrylic resin (28.2 g), Cu_2_O (34 g), Fe_2_O_3_ (5.1 g), ZnO (5.9 g), talcum powder (6.4 g), bentonite (0.2 g), rosin resin (9.4 g), auxiliaries (4 g), and xylenes (2 g). A coating without polyphenol derivative was also prepared and considered as a control. Then, PVC panels were coated with previously prepared paints and immersed in seawater with a depth of 0.2–2 m for six months. Compared with the control paint, coatings with compounds proved to have effective AF activity. A control panel was covered with many marine macroorganisms, such as macroalgae and barnacles, but only biofilms and a few settled barnacles were found on the treated panels. The panels with compounds **47** and **50** were clear of macrofouling [45].

Wang et al. reported the synthesis of three phthalimide derivatives (**52**–**54**, Figure 1) inspired by capsaicin and the incorporation of these compounds at a concentration of 4.8% into a coating composed of acrylic resin (28.2%), Cu_2_O (34%), Fe_2_O_3_ (5.1%), ZnO (5.9%), talcum powder (6.4%), bentonite (0.2%), rosin (9.4%), copper naphthenate (4.0%), and xylenes (2.0%). A coating containing biocide chlorothalonil (4.8%) was used as a positive control. PVC panels (150 mm × 150 mm × 3 mm) containing the prepared coatings were shown to be effective in the prevention of biofouling after nine months of immersion at a depth of 1 m, presenting less biofilm coverage and barnacle larvae than the control panel [46]. 

Based on the structures of natural products, ceramide and paeonol, which display some AF activity, Zheng et al. designed and synthesized the compound *N*-octyl-2-hydroxybenzamide (**55**, Figure 1) [47]. This compound was found to present activity against a broad spectrum of fouling organisms and was further investigated for activity in coatings. The preparation of the AF coating followed the procedure previously reported by the same research group [48]. Briefly, the antifoulant (10%) was placed in a glass beaker with a mixture of acrylic resin (10 g), rosin (5 g), and xylene (10 mL), and the mixture was dispersed uniformly using an ultrasonic grinder and coated on iron plates (100 × 50 × 0.5 mm). Coatings containing ceramide and paeonol were also prepared and used as controls. After one year of immersion, the control coatings were covered in typical fouling organisms, including sludge, diatoms, algae, barnacles, whereas the plate containing the synthesized compound was free of fouling organisms on the surface. Moreover, this compound was submitted to a biodegradation study through the method of shake flask using filtered natural seawater. It was found that compound **55** is highly biodegradable, with a half-life of 6.3 days. Moreover, this compound can be readily decomposed of microorganisms into small molecules, such as carbonate, carbon dioxide, and water, and is therefore potentially harmless for the environment [47].

Beyazkilic et al. reported the incorporation of the natural antifoulant capsaicin (**56**, Figure 1) into a water-based acrylic coating formulation, as well as the commercial biocide dichlofluanid, at two different concentrations (1.5% wt. and 3% wt.) [49]. The authors also studied the effect of coatings composed of a mixture of commercial biocide and natural compound capsaicin. For the preparation of AF paint formulations, the natural antifoulant capsaicin (1.5% wt. and 3% wt.) and the biocide dichlofluanid (1.5% wt. and 3% wt.), as well as a mixture of both AF agents (capsaicin 1.5% wt. + dichlofluanid 1.5% wt. or capsaicin 0.75% wt. + dichlofluanid 0.75% wt.) were added to a mixture of water (~16%) and dispersant (Disperbyk 2080), followed by the addition of pigment titanium dioxide (Kronos 2360) (~19%), acrylic resin (Alberdingk® AC 2403), and additives (BYK 24 as defoamer, BYK 349 as wetting agent, and BYK 7420 as rheological additive). The previously prepared coatings, as well as a biocide-free formulation and a positive control coating (ECONEA^®^-based coating AquaNet^®^ Protect) were applied onto steel substrate and submitted to an antibacterial assay against the gram-negative bacterium *Aeromonas salmonicida*, a Chilean bacterial strain isolated from seawater Atlantic salmon farms, which is responsible for systematic infections and affects the health of Atlantic salmon. After 48 h of incubation, the coating containing 3% capsaicin showed a high reduction in bacterial colony-forming unit (CFU), higher than the coating containing the same percentage of commercial biocide dichlofluanid. However, the coatings with a combination of capsaicin and dichlofluanid at a percentage of 0.75% of each biocide displayed the highest reduction in CFU, which proved the potential of combining biocides. Regarding leaching assays of the prepared coatings in saline water, it was found that, after four weeks of the assay, the cumulative release of capsaicin from the paint with 1.5 wt.% capsaicin was higher, with a faster release rate when compared with the coating containing a mixture of capsaicin 1.5% and dichlofluanid 1.5%, which might be due to the physico-chemical interactions between capsaicin and dichlofluanid in the same paint matrix, decreasing the release rate of capsaicin, and consequently resulting in a longer-lasting AF effect [49].

### 2.2. Rosin-Based Coatings

Rosin is a naturally occurring resin, which is extracted from various species of pine and conifer trees, that consists of a mixture of unsaturated diterpene monocarboxylic acids, such as abietic, palustric, and pimaric acids and their isomers, which impart strong rigidity to rosin due to their phenanthrene ring structure, and therefore, rosin and its derivatives tend to be hard, brittle, and friable materials with relatively low film strength [50]. Rosin is the main binder of CDPs. In contact with seawater, the binder containing the biocide dissolves, gradually releasing the biocide. The lifespan of this type of coating is limited to 36 months or less [31,32,51].

This matrix has been applied by some research groups to assess the AF activity of natural and synthetic compounds, as mentioned bellow. Rosin-based polymers are expected to be environmentally friendly and biodegradable. Additionally, rosin is abundantly available, making it easily accessible for large-scale production.

**Figure 2:**
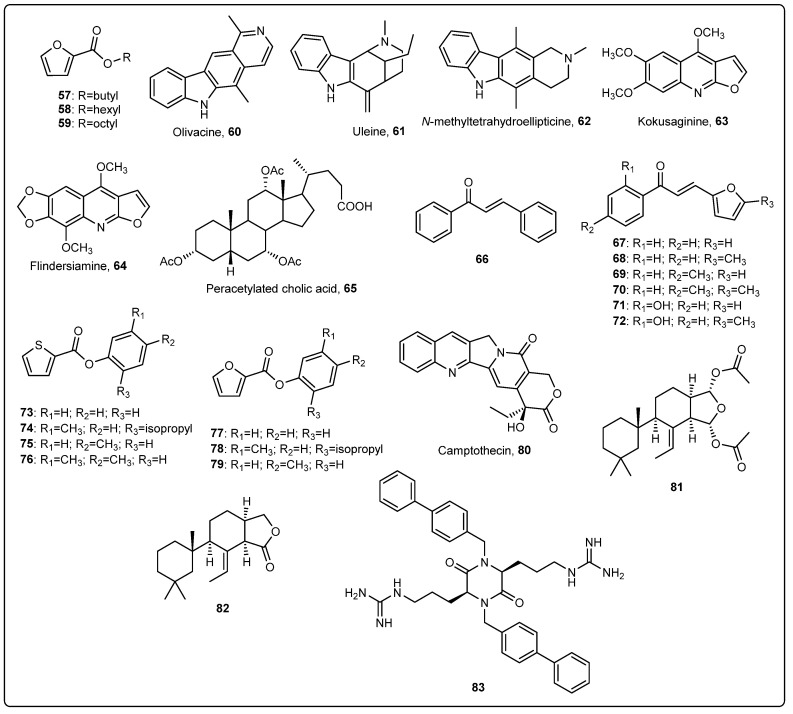
Structures of AF compounds **57**–**83**.

Inspired by the fact that some marine natural products with interesting AF activity contain furan and furanone moieties, such as the natural terpenoid variabilin, isolated from the New Zealand marine sponge *Semitaspongia bactriana* [52], Escobar et al. reported the synthesis of three alkyl 2-furoates (**57**–**59**, Figure 2) obtained by green chemistry procedures and their AF activity when incorporated into a rosin-based paint [53]. The coating formulation included WW rosin (12.5%) and hydrogenated rosin (14.9%) as binders, oleic acid (3.8%) and polyterpene resin (2.3%) as plasticizers, zinc oxide (33.2%) and calcium carbonate (11.1%) as pigments, and toluene (22.0%) as a solvent. Compounds were added in a concentration of 1% wt. A control paint without active compounds was also tested. Acrylic tiles (4 × 12 cm^2^) were coated with the previously prepared coatings and immersed in the sea for 45 days. After this time, it was found that coatings containing alkyl 2-furoates were less fouled by macrofouling species than negative control paint, and coating containing compounds butyl 2-furoate (**57**) and hexyl 2-furoate (**58**) were more active than AF paint containing octyl 2-furoate (**59**) [53].

Pérez et al. reported the isolation and AF activity evaluation of five natural alkaloids from trees of the Atlantic rainforest, namely olivacine (**60**), uleine (**61**), and *N*-methyltetrahydroellipticine (**62**) from *Aspidosperma austral*, and the furoquinoline alkaloids kokusaginine (**63**) and flindersiamine (**64**) (Figure 2) from *Balfourodendron riedelianum* [54]. Alkaloids **62** and **63** displayed the most active anti-settlement activity against mussel *M. edulis platensis* [54]. Compounds **60**–**64** were further incorporated into a coating and submitted to a field test. For the preparation of base paint, the binders hydrogenated rosin (14.9%) and WW rosin (12.5%) and plasticizers oleic acid (3.8%) and polyterpene resin (2.3%) were dispersed in toluene. Then, the pigments zinc oxide (33.2%) and calcium carbonate (11.1%) were added and dispersed for 24 h. One portion of paint was used as a negative control, and each alkaloid was incorporated into the other parts of the base paint at 250 ppm. Paints were applied to acrylic tiles (4 × 10 cm^2^), which were further immersed in the sea for 45 days. Coatings with compounds **62**–**64** presented much fewer fouled organisms, including algae, bryozoans, tunicates, and crustaceans, than the negative control coating, whereas coatings containing compounds **60** and **61** were less effective [54].

Peracetylated cholic acid (**65**, Figure 2), a bile acid derivative isolated from the Patagonian sponge *Siphonochalina fortis* and easily obtained from chemical synthesis from cholic acid, was shown to present anti-settlement activity against the mussel *Mytilus edulis platensis*, and was further submitted to field assays, through incorporation in a rosin-based coating [55]. Coating formulation included rosin (22%) as a binder, oleic acid (4.6%) as a plasticizer, zinc oxide (34.6%) and calcium carbonate (11.1%) as pigments, and a mixture of xylene/white spirit/methyl isobutyl ketone (1:1:0.5, 25.4%) as a solvent. Compound **65** was dissolved in methanol and incorporated into the matrix paints at 0.6% concentrations. The AF coatings were applied to acrylic tiles (4 × 12 cm^2^) and further immersed in the sea for three months. An uncoated panel, as well as a panel coated with the matrix paint without antifoulant were also immersed and used as negative controls. After this time, panels containing coating with compound **65** presented much fewer fouling organisms from several species, compared with the negative control panels [55].

Considering that several natural and synthetic chalcones and furylchalcones have been reported to display antimicrobial activity and aiming to study their effect on the prevention of marine biofouling, Sathicq et al. synthesized the non-substituted chalcone (**66**) and six furylchalcones (**67**–**72**), which were incorporated into a coating to evaluate their AF activity [56]. Rosin-based paint was prepared by solubilizing the binders’ hydrogenated rosin (14.9%) and WW rosin (12.5%) and the plasticizers’ polyterpene resin (2.3%) and oleic acid (3.8%) in toluene, followed by the adding of pigments of calcium carbonate (11.1%) and zinc oxide (33.2%). One part of the paint was used as a control and chalcones were incorporated into the other portions of the base paint (1.25 mmol chalcone/100 g of paint). Paints were applied to acrylic panels (4 × 12 cm^2^) which were further immersed in the sea at a depth of 0.5 m for 45 days. Compared with the control paint, the coatings containing chalcones were able to inhibit the fouling of macro- and microfouler species during the field assay and, generally, the furylchalcones were shown to be more active than the non-substituted chalcone [56].

Taking into account that several compounds containing furan of thiophene groups have been reported to play a significant role in AF activity, the synthesis of a series of phenyl esters containing furan or thiophene rings was performed by Escobar et al. and eight compounds with high yields were obtained [57]. Considering the AF potential of molecules containing furan and thiophen rings, these compounds were hypothesized to display AF activity and were incorporated into a rosin-based coating at 1% wt., prepared as described previously by the same research group [53]. Painted panels were submerged in the sea for three months, and it was found that, from the eight compounds synthesized in this study, seven compounds (**73**–**79**, Figure 2) were shown to inhibit the settlement of fouling organisms, since the coated panels were much less covered than the control paint [57].

Hao et al. studied the AF performance of a rosin-based coating in which the natural compound camptothecin (**80**, Figure 2) at a concentration of 10% was added for the prevention of marine biofouling [58]. A paint composed of rosin (10%), chlorinated polyether resin (14%), Fe_2_O_3_ (5%), bentonite (1%), zinc oxide (15%), talcum powder (10%), and xylene (35%) was prepared and then was used to coat six different materials frequently used for the construction of underwater sensor housings, namely 316 L stainless steel, TC4 titanium alloy, 7075 aluminium alloy, polyoxymethylene, PVC, and Teflon. After six months of immersion in the sea at a depth of 1 m, all materials were shown to be less fouled than materials without the previously prepared coating, and the AF performance on the plastic materials were shown to be better when compared with the metal materials [58].

Prieto et al. reported the extraction of two diterpenoids, 9,11-dihydrogracillin A (**81**) and 9,11-dihydrogracillinone A (**82**) (Figure 2), from the sponge *Dendrilla antarctica* and their AF evaluation after incorporation into a rosin-based coating [59]. A base paint was formulated with hydrogenated rosin (14.9%) and WW rosin (12.5%) as binders, oleic acid (3.8%) and polyterpene resin (2.3%) as plasticizers, zinc oxide (33.2%) and calcium carbonate (11.1%) as pigments, and toluene as a solvent, as previously reported by the same research group [54]. Compounds **81** (100 mg%) and **82** (25 mg%) were further added to the base paint. Acrylic panels (4 × 12 cm^2^) were coated with paints containing diterpenoids, as well as a control paint without active compounds. The painted panels were submersed in the sea at a depth of 0.5 m for three months, and then the coverage area for the different fouling organisms was analyzed. It was found that panels coated with diterpenoids showed a marked reduction in the covered area of fouling organisms compared with the control paint [59].

Cahill et al. reported the high-scale synthesis of a cyclic dipeptide based on the structure of the natural motif 2,5-diketopiperazine, which has previously proved to display high AF potential against macrofouling ascidians, bivalves, tubeworms, seaweeds, and barnacles, as well as some microfouling bacteria and algae [60], and the incorporation into a rosin-based coating [61]. The coating was prepared by dissolving rosin (57.5 g) and an acrylic copolymer binder (Tires 4015–SS–50, 25 g) in xylene (18.75 g). Then, lecithin (2.5 g), iron (III) oxide (40 g), benzyl phthalate (10 g), barium sulfate (165 g), bentonite (5 g), and silicon dioxide (2.5 g) were added to the mixture. Compound **83** (Figure 2) was added at 1% wt. to the mixture. After 2 and 11 weeks of immersion of PVC cylinders, coating with 1% wt. compound displayed lower fouling organisms than control coating without an active compound. Nevertheless, after seven months of immersion, there was no difference between the two coatings. These results can be explained by the fact that compound **83** was found to be at the surface of the coating film using time-of-flight secondary ion mass spectrometry (ToF-SIMS) analysis. Moreover, the environmental stability of compound **83** was assessed through theoretical biochemical oxygen demand and analytical quantification using a closed bottle method, showing that the half-life of compound **83** ranges from 13.4 to 16.2 days.

### 2.3. Coatings Based on Synthetic Biodegradable Polymers

**Figure 3:**
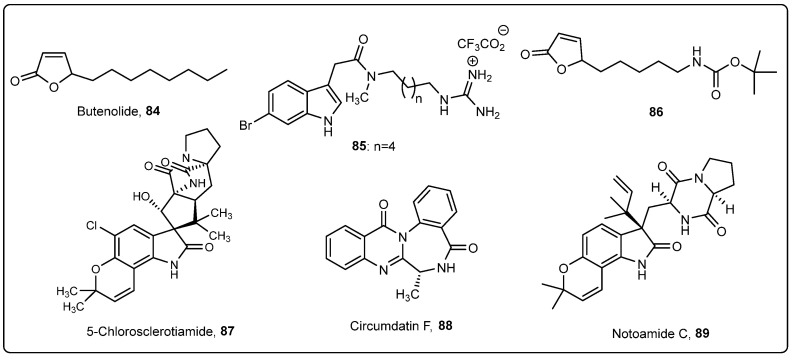
Structures of AF compounds **84**–**89**.

Several butenolides (isolated from the crude extract of a marine *Streptomyces* strain obtained from deep-sea sediments, as well as the synthetic butenolide 5-octylfuran-2(5*H*)-one (**84**)) have shown AF activity against cyprid larvae of the acorn barnacle *Balanus amphitrite* [62]. Moreover, compound **84** was shown to display antibiofilm activity [63]. Considering this, Chiang et al. reported the incorporation of the antifoulant butenolide (**84**, Figure 3) in a coating based on a biodegradable synthetic polymer poly(lactic acid)-based polyurethane (PLA-PU50) [64]. The PLA-PU50 polymer was synthesized by the polyaddition of isophorone diisocyanate (IPDI, 16.7 mmol) and Poly(L-lactide) diol (PLA, 2.5 mmol) using THF as solvent, under heating for 1 h, yielding a prepolymer. Then, 1,4-butanediol (BDO, 14.2 mmol) and dibutyltin dilaurate (DBTDL, 0.2 wt.%) were added as the chain extender and catalyst, respectively, and the mixture was allowed to react under heating for 3 h, yielding the polymer. After the synthesis of the polymer, compound **84** was added in different concentrations (1%, 5%, 10%, and 20%), using xylene as a solvent. The solution was applied to the surface of PVC panels (53 × 125 mm^2^), which were further immersed in seawater at a depth of 1 m for three months. The research group also prepared coatings combining the biodegradable polymer with binder rosin, with PLA-PU50: rosin ratios of 2:1, 1:1, and 1:2. Butenolide was added in a concentration of 10% to the PLA-PU50: rosin coatings. After three months of contact with seawater, panels with 10% of butenolide and a PLA-PU50: rosin ratio and panels with 20% of butenolide without rosin showed similar performance, with a non-covered area of approximately 20%. As in the previous study, these results showed that the incorporation of rosin into the coatings increased the self-renewal rate of the polymer and facilitated the long-term release of butenolide from the coating, which increased the AF efficiency [64].

In a work performed by Pan et al., a biodegradable polyurethane polymer composed of PLA or poly(lactide-co-glycolide) (PLGA) as soft segments and different contents of triisopropylsilyl acrylate (TSA) as pendant groups was synthesized through a thiol−ene reaction and polyaddition and then was used for the preparation of a coating with the antifoulant butenolide **84** (Figure 3) using THF as a solvent [65]. Coated PVC panels were immersed in the sea at a depth of 0.5 m for three months. The marine field test shows that coating with 10% of antifoulant and with a higher content of TSA showed good AF ability for more than three months. Moreover, the authors also showed that butenolide (**84**) was released from the coating in a continuous manner with a controlled rate as the polymer degraded in seawater [65].

Compound **85** (Figure 3), a synthetic analogue of marine natural antifoulant phidianidine A, synthesized by Labriere et al., was shown to display potent anti-settlement activity against *Amphibalanus improvisus* cyprids and was submitted to a field test [66]. For the preparation of the coating, the biodegradable polymer poly(ε-caprolactone-co-δ-valerolactone) (80:20) was synthesized according to previous experience [67] and solubilized in xylene (mixture of isomers) (1:1). Then, the compound was dissolved in methanol and added to a polymer solution at a compound concentration of 0.1 mg/L. PVC panels were coated and immersed in the sea for 84 days. Uncoated PVC panels and panels coated with paint without bioactive compound were used as negative controls. The growth of microorganisms on the coated plates was assessed using confocal laser scanning microscopy (CLSM). The coating incorporating the bioactive compounds displayed lower coverage and biovolumes of bacteria and microalgae than the controls, which suggested the potent AF activities of this compound against marine microfoulers [66].

In order to increase the melting point and to remove the foul smell of butenolide **84**, a synthetic derivative **86** (Figure 3) was obtained by Chiang et al. [68]. AF assays showed that the synthetic compound had similar activity compared with butenolide. Both compounds, at concentrations of 1%, 2.5%, 5%, and 10%, were incorporated into coatings composed of the polymer poly(ε-caprolactone)-polyurethane (PCL-PU) [69], using xylene as a solvent. PVC panels (53 mm × 125 mm) were coated with the previously obtained paint and immersed in seawater at a depth of 1 m for two months. The synthetic derivative showed similar AF performance compared with the natural antifoulant butenolide. Moreover, an assay to evaluate the release of compound **86** from the coating was performed and compared with butenolide **84** and showed that the release of compound **86** from the coatings at four concentrations was much lower than that of butenolide after one month [68].

Gao et al. reported the influence of alkaloids 5-chlorosclerotiamide (**87**), circumdatin F (**88**) and notoamide C (**89**) (Figure 3), produced by the deep-sea-derived fungus *Aspergillus westerdijkiae*, on the biofilm formation of marine fouling microbial communities [70]. These compounds were incorporated into coatings composed of 0.2 g of each compound and 45% degradable polyurethane (10 mL) in xylene, which were then painted on PVC plates (10 cm × 10 cm). Control plates with only 45% degradable polyurethane (10 mL) in xylene were also assayed. After immersion in seawater at a depth of 1 m for one month, the control plates were fully covered with biofilm, while plates treated with the three compounds showed distinct AF activity against microfouling. Compound circumdatin F (**88**) showed the strongest AF activity against fouling bacteria, followed by notoamide C (**89**) and 5-chlorosclerotiamide (**87**). The average microbial settlement ratios in the control and treatment groups with compounds **87**, **88**, and **89** were 97%, 76%, 29%, and 36%, respectively [70].

### 2.4. Other Coatings

**Figure 4:**
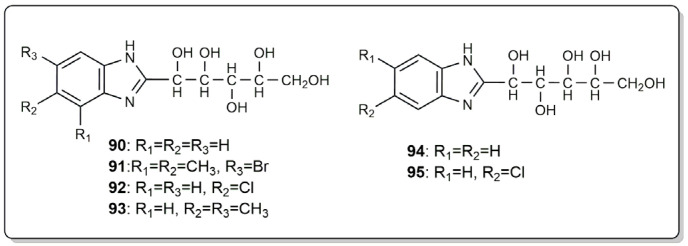
Structures of AF compounds **90**–**95**.

Liu et al. reported the development of a novel coating technique for the loading of the environmentally friendly biocide capsaicin (**56**, Figure 4), for marine AF applications [71]. At two different concentrations (1% wt. and 2% wt.), capsaicin was mechanically blended with high density polyethylene (HDPE) powder, a thermoplastic polymer made from petroleum. However, HDPE is easily recyclable, being a cost-effective, environmentally responsible plastic. HDPE is known for having a high strength to density ratio as well as great resistance to corrosion in maritime environments [72]. Thanks to its high malleability, rigid strength, and corrosion resistance, HDPE is the perfect combination of strength, cost-efficiency, and environmental friendliness. These coatings were deposited by flame spray on mild steel plates, using acetylene as the fuel gas. A control coating with only HDPE and an uncoated plate were used as negative controls. The microstructural features of HDPE–capsaicin coatings were characterized. The capsaicin in coatings showed significant changes in the morphology and grain size compared with the starting coating material. The AF performance of the coatings was assessed against marine gram-positive *Bacillus* sp. (MCCC No.1A00791) and gram-negative *Escherichia coli* (ATCC 25922), as well as against diatom *Phaeodactylum tricornutum*. After 48 h of exposure of coated and uncoated plates to the bacterial strains, the coating composed of 2% wt. of capsaicin was able to decrease by almost 100% the growth of *Bacillus* sp. and *E. coli*, whereas the coating composed of 1% wt of capsaicin was able to decrease the growth of *Bacillus* sp. by almost 100% but did not present the same efficiency for *E. coli*. Uncoated plates or those coated only with HDPE were not able to inhibit bacterial growth. The coating containing 2% of capsaicin was also able to reduce the adhesion of *Phaeodactylum tricornutum* after one week of incubation [71].

Sallam et al. reported the synthesis of a series of benzimidazole derivatives, their incorporation into a marine coating, and the evaluation of the decrease in the biofilm bacterial number [73]. The marine paint was based on 25 g of oil binder material, 10 g of iron oxide, 24 g of zinc oxide, 13 g of complementary piment, and 38 g of xylene. Compounds were incorporated at a concentration of 2%. After the immersion of steel panels coated with the previously prepared coatings for two and four weeks, it was found that coatings containing benzimidazole derivatives **90**–**95** (Figure 4) showed a significant decrease in the biofilm coverage in comparison with the negative control and positive control panels, which suggests that these compounds were useful for the inhibition of the marine bacterial growth [73]. Moreover, the results of the chemical parameters (pH value, alkalinity, dissolved oxygen, oxidizable organic matter, important nutrient salts which are the dissolved inorganic forms of nitrogen (NO_2_^−^, NO_3_^−^, and NH_4_^+^), dissolved inorganic phosphate (PO_4_^3−^), and silicate (SiO_3_^−^) of the surrounding seawater) indicated that all coated panels with marine paint formulations containing compounds **90**–**95** did not present any bad effects on the seawater surrounding the coated panels. Therefore, it can be inferred that these compounds will not cause pollution to the marine environment and subsequently to marine organisms.

## 3. Commercial Coatings Containing Natural and Synthetic Antifoulants

### 3.1. Acrylic-Based Coatings

Commercial two-component acrylic (AV)-based coating was used to evaluate the anti-settlement activity of three nature-inspired synthetic compounds (**96**–**98**) as described in the following.

**Figure 5:**
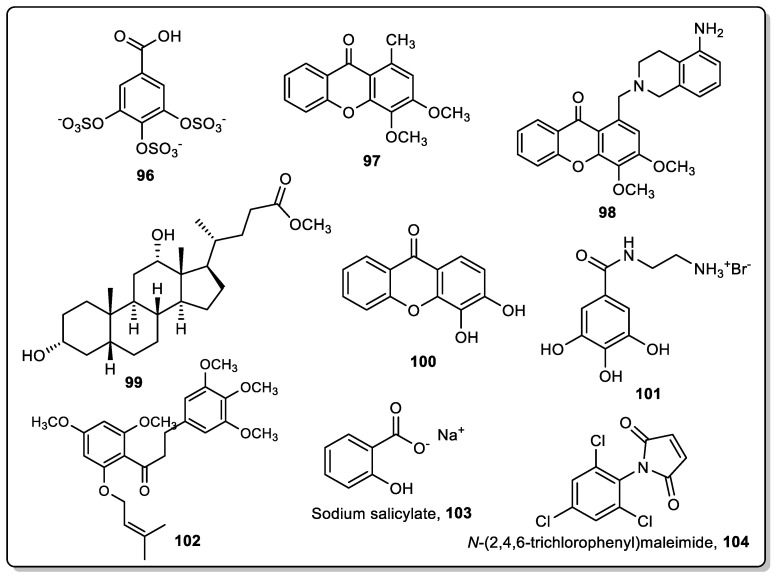
Structures of AF compounds **96**–**103**.

Based on the AF potential of zosteric acid, a sulfated phenolic acid isolated from the seagrass *Zostera marina* [74], the nature-inspired antifoulant gallic acid persulfate (GAP, **96**) (Figure 5) was synthesized and evaluated for AF activity, showing high potential to inhibit the settlement of mussel *Mytilus galloprovincialis* larvae [75]. Later, the anti-settlement activity of the compound was tested in an AV coating system [76]. The immobilization of this compound followed two strategies: conventional direct incorporation and chemical immobilization promoted by the addition and blending of the trimethylolpropane triaziridine propionate crosslinker (TZA). The direct incorporation allowed for the assessment of the feasibility of the new synthesized **96** as an AF agent in conventional release AF coating systems, while the chemical immobilization strategy demonstrated the potential of **96** to be grafted in polymeric coating systems, thus promoting long-lasting effects. The proportions of volume of the paint components’ base/curing agent used were 3/1 for the AV wet system, in accordance with the instructions provided by the coating components’ suppliers. The bioactive compound was directly added and blended with the coating components to allow a final concentration in the final coating of 0.5 and 1%. After coating polystyrene 24-well plates with the prepared coatings, or with a control paint without bioactive compound, an AF bioassay with the larvae of the mussel *M. galloprovincialis* was performed in the laboratory. This anti-settlement study showed that, despite a settlement of 15% still being observed on wells coated with AV coating containing **96** chemically immobilized, the chemical immobilization with TZA decreased nearly 40% of the larvae adhesion compared with AV coating with **96** directly incorporated. 

Some natural xanthones obtained from marine sources have been shown to display anti-settlement and antimicrobial activities [77,78]. Moreover, several nature-inspired xanthones obtained by synthesis, such as compounds **97** and **98** (Figure 5), showed effective activity in the inhibition of the settlement of *M. galloprovincialis* mussel larvae. Later, xanthones **97** and **98** were incorporated in the same commercial AV-based coating [79]. Xanthone **97** was previously dissolved in dichloromethane and further added and blended into the AV coating components in the exact amount to yield a 0.55% of xanthone **97** in the wet formulation system. Similarly, a previous dissolution of xanthone **98** in methyl pyrrolidone was added and blended into the AV coating components, yielding a final concentration of 1% in the AV wet system. For these AV-based coatings, 70% and 80% inhibition of the *M. galloprovincialis* larval settlement was observed in wells coated with coatings containing xanthones **97** and **98**, respectively, in contrast to the compound-free AV-based coating in which a larval settlement of 50% was observed.

### 3.2. Polyurethane-Based Coatings

Polyurethane (PU) represents a class of segmented copolymers, composed of a “soft” segment, typically a low molecular weight polyol, and a “hard” segment, made of diisocyanate and a chain extender. The “soft” segments impart elastomeric behavior to the PU, while the “hard” segments are responsible for mechanical resilience. Additionally, the hard segments contain urethane and/or urea bonds, which are capable of forming hydrogen bonds. This characteristic confers PU good wettability, as well as high strength and elasticity, unique properties that make PU a polymer of choice in the biomedical field, as well as in the marine sector [80,81,82]. Despite the exceptional characteristics mentioned above, isocyanate used in the synthesis of traditional PU presents toxicity, volatility, and a potential risk to human health (such as dermatitis and asthma). Furthermore, the production of isocyanate involves the use of phosgene, a highly toxic substance, further exacerbating environmental and health hazards. Additionally, isocyanates are susceptible to water, leading to increased expenses in production, transportation, and storage [83].

Commercial PU-based marine paint was used to evaluate the anti-settlement activity of their synthetic compounds (**96**, **97**, **99**, **100**–**102**) as described in the following.

Based on the AF potential of some marine-derived steroids [14,15], Neves et al. reported the synthesis of a series of bile acid derivatives and AF evaluation against some macro- and microfouling species [84]. Considering the results obtained, compound **99** (Figure 5) was selected for incorporation into a PU-based marine paint composed of two components, the base resin and the curing agent. For the preparation of the optimized formulation, the bioactive derivative was dissolved in *N*-methylpyrrolidone at a bioactive derivative/solvent weight ratio of 13.95, and this solution was blended into the paint components, the base and curing agent (ratio of 9/1). The bioactive derivative was present at a content of 0.58 wt.% in the final prepared coating. A control paint without bioactive compounds and a paint with commercial biocide ECONEA^®^ were also prepared. The coatings were used to also coat 24-well plates and an in vivo anti-settlement assay using larvae of mussel *Mytilus galloprovincialis* was performed. Compared with the coating with the commercial biocide ECONEA^®^, coating containing compound **99** were shown to be more effective in inhibiting the larval settlement after 40 h of exposure. 

Compound **96** (Figure 5), besides AV coatings, was also incorporated in a PU-based system composed of two components, the base resin and the curing agent, in a 2/1 ratio, following conventional incorporation and chemical immobilization with TZA [76]. This matrix supported a final 2 wt.% content of this compound, higher than the AV coating. After coating 24-well plates with the prepared coatings, or with a control paint without a bioactive compound, an AF bioassay with the larvae of the mussel *M. galloprovincialis* was performed, which showed that (i) the direct incorporation of compound **96** did not succeed as the larval settlement on wells coated with this formulation was similar to the wells coated with the control paint and (ii) the PU coating formulated with **96** and TZA exhibited high anti-settlement activity (only 5% of larvae were settled on) in contrast to the control paint and the coating containing the compound directly incorporated into the paint. Leaching studies were performed by immersing PVC plates (3.5 × 6 cm) coated with the prepared coatings in artificial seawater for 45 days. The percentage of **96** released from PU coatings with **96** chemically immobilized was almost five times lower (4%) than that from PU coatings with **96** directly incorporated (nearly 20%), which explains the AF efficacy of the TZA formulation [76].

The same group also described the immobilization of the synthetic 3,4-dihydroxyxanthone (**100**, Figure 5) with AF activity in the same two components of PU-based marine paint and following both strategies [21]. The chemical immobilization of this compound was based on a pre-functionalization approach with an isocyanate. The 4,4-diphenyl diisocyanate-monomeric (MDI) (isocyanate content of 33.4 ± 0.1%) was added dropwise to compound **100** in tetrahydrofuran, at 40 °C, under stirring and inert atmosphere conditions into a three-necked round bottom flask. Solutions of compound **100** and its isocyanate derivative were further added and blended into the paint PU components (base/curing agent 2/1) in the exact amounts to yield the desirable agent contents in the final coating of 2 wt.%. A control paint without biocides and a PU-based marine coating containing the commercial biocide Econea^®^ directly and chemically immobilized were also prepared for comparative purposes. The prepared formulations were used to coat polystyrene 24-well plates and an AF assay with the larvae of the mussel *M. galloprovincialis* was performed. It was found that coatings containing compound **100** directly and chemically immobilized into the marine coating were less attached with mussel larvae than control coating or the marine coating containing commercial biocide Econea^®^. PVC plates were also coated to perform leaching studies. While a premature leaching was observed for compound **100** from PU coatings containing compound **100** directly added, compound **100** was not detected in leached waters that were in contact for 45 days with plates coated with PU coatings containing this compound chemically immobilized, demonstrating the potential of this formulation for a non-biocide release strategy in commercial marine coatings.

Later, Neves et al. reported the synthesis of other gallic acid derivatives and their AF evaluation [18]. Among them, compound **101** (Figure 5) showed high potential to prevent the settlement of the mussel *M. galloprovincialis* and was incorporated as an additive at a content of 2 wt.% into the previously reported two components commercial PU-based coating. For the preparation of the coating, compound **101** was dissolved in *N*-methylpyrrolidone at a ratio of 0.41 (**101**/solvent weight), which was further added and blended into the PU paint (base/curing agent ratio = 11/1). The previously prepared paint, as well as a control paint without biocide, were used to coat a 24-well microplate for the anti-macrofouling evaluation of the compound **101**-based coating. Compared with the control paint, a decrease in the settlement in the compound **101**-based coating was found, highlighting the role of compound **101** as an AF additive for PU-coatings [18]. Moreover, considering the potential of this compound to prevent the biofilm formation in a solution as well as to reduce a pre-formed biofilm produced by the marine bacterium *Pseudoalteromonas tunicata*, this compound was later submitted to an evaluation of its performance as an anti-biofilm agent in surface coatings [85]. For this assay, the compound **101** was incorporated conventionally and through the use of the crosslinker TZA at different concentrations, close to 1.0 and 2.0 wt.%, into the same commercial PU-based marine paint. For the conventional preparation of PU-based formulations, the compound was firstly dissolved in *N*-methyl pyrrolidone with a compound/solvent weight ratio of 0.38, and then was added and blended into the PU-based paint components (base/curing agent ratio of 9/1) to obtain coatings with a content of bioactive compound of 1 and 2 wt.%. After the preparation of coatings, glass substrates were coated and the formation of *P. tunicata* biofilms was assessed under hydrodynamic conditions to mimic the marine environment. The coating containing 2 wt.% of the bioactive compound **101** with TZA crosslinker provided the best long-term performance [85].

Vilas-Boas et al. also reported the incorporation of the AF xanthone **97** (Figure 5), prior to being dissolved in dichloromethane in a PU-based paint (proportion of 2/1 of base/curing agent) at a final concentration of 2% wt. and the evaluation of the anti-settlement activity against mussel *M. galloprovincialis*. The PU marine coating containing xanthone **97** was shown to be more effective against the settlement of the mussel larvae after 40 h of incubation (10% larval settlement) than the control PU marine coating (35% larval settlement) [79]. As expected from the low water solubility, xanthone **97** presented a leaching value lower than 2%, after 45 days in artificial seawater, indicating the potential to generate long-lasting PU-based coatings.

Based on the AF activity of prenylated dihydrochalcone **102** against the settlement of *M. galloprovincialis* mussel larvae (EC_50_: 9.04 µM), Pereira et al. performed the incorporation of this compound at 1%, 2%, and 5% into a commercial PU-based coating (proportion of 7/1 of base/curing agent) [86]. The evaluation of the anti-settlement activity against *M. galloprovincialis* mussel larvae after 40 h of exposure confirmed the AF potential of PU coating containing compound **102**, particularly for 2% and 5% concentration, which presented lower than 20% of larval settlement, whereas control coating without a bioactive compound presented more than 70% settlement. The seawater of the wells containing coatings with 5% of compound **102** was analysed by HPLC after 40 h of experiments to infer the presence of the compound in seawater and to better understand the mussel inhibition activity. Nonetheless, the chromatographic signal of the active compound was not detectable, and for this reason, it can be concluded that the leaching of compound **102** from the coatings was not considerable after 40 hours, and the AF activity could be due to the influence of the surface of the coating containing the compound. [86].

### 3.3. Silicone-Based Coatings

Silicone-based coatings, specifically PDMS elastomers, are known for their low surface energy and low elastic modulus, making them the foundation of most fouling release coatings (FRC). Coatings with a low surface free energy, between 20 and 30 mN/m, have optimal fouling release performance since they allow minimal bioadhesion. This is because these coatings are engineered to reduce the interactions with biomolecules, by eliminating the capacity for strong polar interactions (like hydrogen or ionic bonding). By relying solely on dispersive interactions, biomolecules can only weakly adhere to these surfaces, facilitating their removal.

The main proposal of FRCs is to minimize the adhesion between fouling organisms and the surface, so biofouling that loosely adheres to these surfaces can be effortlessly removed through the shear forces of water flow or by a gentle mechanical cleaning. An initially fouled FRC-coated surface is able to self-clean at different velocities. Moreover, the smoothness of FRCs enables them to reduce the drag of the vessel and therefore reduce fuel consumption and greenhouse gas emissions. The reported lifetime in service of FRCs is typically 5−10 years [4,32,87]. However, these characteristics also result in the poor adhesion of PDMS coatings to substrates and low resistance to mechanical damage, as well as inadequate static fouling resistance, limiting their applicability in the marine sector [26,88,89].

Some researchers also tested the efficacy of synthetic AF compounds **96**–**99** when added to silicone-based coatings.

Neves et al. incorporated compound **99** (Figure 5) into a silicone-based commercial coating, room-temperature-vulcanizing polydimethylsiloxane (RTV11, MOMENTIVE), composed of a base component and a curing agent [84]. The bioactive derivative was incorporated at contents of 0.58 wt.%. For the RTV-PDMS coating formulation, the bioactive derivative was previously dissolved in *N*-methylpyrrolidone at a bioactive derivative/solvent weight ratio of 17.17, and this solution was blended into the paint components at a base/curing agent ratio of 200/1. Further, 24-well microplates were coated with the coating and submitted to an anti-macrofouling evaluation against the settlement of *M. galloprovincialis* mussel larvae. A compound-free RTV-PDMS coating was used as a negative control and an RTV-PMDS coating containing biocide Econea^®^ was used as a positive control. After 40 h of incubation, for coatings containing Econea^®^ or bile acid derivative **99**, no mussel larval settlement was observed, whereas some settlement was found for negative control coating [84].

Vilas-Boas et al. also added the persulfated gallic acid GAP (**96**) into a marine silicone-based coating (PDMS), following conventional incorporation and chemical immobilization [76]. Compound **96** demonstrated good compatibility with this silicone-based matrix. PVC plates coated with the prepared PDMS coatings were also immersed in artificial seawater for 45 days to quantify compound **96**’s release to water. The chemical immobilization of GAP in this silicone-based coating decreased the release of **96** from 21% (observed for direct immobilization) to 11%. However, this coating provided non-informative results regarding the AF effect of compound **96** due to a high anti-settlement effect observed for both formulations, i.e., with and without compound **96**. To overcome this masked effect from the intrinsic properties of the marine PDMS coating, complementary assays with RTV-PDMS-based non-marine coating were performed following both conventional direct incorporation and chemical immobilization. GAP (**96**) also demonstrated good compatibility with this silicone-based matrix [76]. The anti-settlement studies with this new coating allowed for the understanding that the presence of compound **96** in the formulation significantly improved the anti-settlement activity, compared with the control formulation (RTV-PDMS without **96**). This effect was even more pronounced when compound **96** was chemically immobilized as no larvae was found to have settled. Leaching studies were later conducted with RTV-PDMS coatings with **96** directly incorporated**,** and an analytical study of the leached waters demonstrated the potential of RTV-PDMS to generate long-lasting coatings with compound **96**, as only 0.35% of compound **96** was released after 45 days [90]. 

Xanthones **97** and **98** were also incorporated by the same group in PDMS (HEMPASIL X3+87500) and in a non-marine coating system RTV-PDMS [79]. Xanthone **97** was previously dissolved in dichloromethane to provide solutions with compound contents of 12.58 and 6.81 wt.%, which were further added and blended into PDMS and RTV-PDMS coating components, respectively, and in the exact amounts to yield the desired xanthone **97** contents of 0.52–0.53 wt.% in those wet formulation systems. Similarly, a previous dissolution in methyl pyrrolidone was performed for xanthone **98**, resulting in solutions with xanthone **98** contents of 9.35 and 9.24 wt.%, which were further added and blended into the PDMS and RTV-PDMS coating components, respectively, yielding a final concentration of 0.53–0.55 wt.% in the formulation systems. The proportions of volume of the paint components base/curing agent used were 17.8/2.2 for the PDMS marine paint, and 199/1 for the RTV-PDMS wet systems, in accordance with the instructions provided by the coating components suppliers. A high anti-settlement effect was observed in the wells coated with the PDMS compound-free formulation, providing non-informative results regarding the AF effect of these two AF xanthones after incorporation in the silicone matrix. The same results caused by this non-stick coating were also observed in previous anti-settlement assays with the compound **96** [76]. Nevertheless, it was found that, after 45 days of the leaching assays, xanthones **97** and **98** were leached from the PDMS-based marine coatings by approximately 17% and 25%, respectively, whereas the same compounds showed a leaching value lower than 2% from the PU-based coatings, as reported in Section 3.2. These results may indicate a possible short time effect of these compounds in the PDMS coating [79].

With RTV-PDMS coating, it was possible to observe significant differences in the larval settlement on the wells coated with xanthones **97**- and **98**-based-RTV-PDMS coating compared with the compound-free RTV-PDMS coatings. In fact, 100% inhibition of *M. galloprovincialis* larval settlement was observed on both RTV-PDMS-based coatings containing xanthones **97** and **98**, after 40 h, in contrast to the compound-free RTV-PDMS coatings which exhibited 10% larval settlement [79].

### 3.4. Epoxy Coatings Containing AF Compounds

Epoxy resins represent a crucial category of polymer materials, distinguished by the presence of multiple three-membered rings, often referred to as epoxy, epoxide, oxirane, or ethoxyline groups [91]. Epoxy resins are high molecular weight binders which compose the contact leaching coatings. Contact leaching coatings are known as hard AF paints, in which high amounts of biocides can be incorporated. Considering that the binder is not soluble in seawater, the thickness of the coatings remains constant as the antifoulants are released. These AF coatings present a short efficiency duration between 12 and 24 months since, as the exposed toxicant particles are deeper in the paint film, the toxicant release rate gradually decreases with time, and the protection becomes increasingly inefficient [4,32,51]. However, epoxy coatings are vulnerable to long-term corrosion because oxygen and aggressive species can penetrate through the pores in the coating layers, reaching the metallic substrate. Epoxy coatings provide a recognized protection solution for metals due to their unique combination of properties, such as ease of processing, low curing shrinkage, excellent mechanical properties, and superior chemical and corrosion resistance [92]. When cured, most epoxy resins form amorphous thermosets that exhibit exceptional mechanical strength and toughness. They also offer remarkable resistance to chemicals, moisture, and corrosion, along with excellent thermal, adhesive, and electrical properties. Additionally, these resins present low emissions of volatile organic compounds, and are therefore environmentally safe. Due to these outstanding performance traits, combined with high formulation versatility and cost-effectiveness, epoxy resins have become widely favored for various applications, including bonding, structural uses, and protective coatings [91].

Tonelli et al. reported the incorporation of two compounds with AF potential, namely sodium salicylate (**103**) and *N*-(2,4,6-trichlorophenyl)maleimide (TCPM, **104**) (Figure 5) into a commercial epoxy coating [93]. These compounds were encapsulated into halloysite nanotubes (HNTs) before being mixed into the paint in an attempt to prolong their protection and prevent a fast release from the paint. After the encapsulation of active molecules, the HNTs were incorporated into the epoxy paint Penguard HB, which is composed of two components, component A (epoxy resin) and component B (amide-based curing agent). HNTs loaded with active compounds **103** and **104** (10% percentage of loaded HNTs in final composite) were incorporated into component A, and then component B (4:1 component A/component B volume ratio). After the preparation of the coating, films with a thickness of 112.8 μm were prepared. Coatings containing active compounds without being encapsulated into HNTs were also prepared for comparative purposes, particularly in the study of the advantage of using HNTs in the delivery of active compounds from the paint. A control coating with HNTs without active compounds was also prepared. The dispersion of nanotubes into the paint was evaluated after the preparation of a spread film of the paint by means of Scanning Electron Microscopy (SEM). Considering that no agglomerates were found when scanning the samples, it suggests that the nanotubes were well dispersed in the paint. The evaluation of the dynamic water contact angle showed a large hysteresis for all the investigated samples, with the sample with compound **103** having the lowest value of hysteresis. This value can be explained by the lowest value of the advancing contact angle in comparison with the other two coating systems, which can be associated with the presence of compound **103**, which introduces hydrophilic moieties on the surface. The coatings were submitted to an AF assay to evaluate the ability of these coatings to inhibit the attachment of the marine bacteria *Vibrio natriegens*. After 7 days of incubation, about 38% of the total exposed surface of the control paint containing only HNTs was covered by bacteria, while less than 1% of the surfaces of paints containing HNTs with sodium salicylate (**103**) or TCPM (**104**) were attached by the bacteria. The evaluation of the kinetics of release of compounds **103** and **104** from the composites revealed that the release of AF agents from HNTs into the water was very slow and most of the active molecules remained trapped into the matrix, prolonging the protective effect against marine fouling [93].

Among the 104 AF compounds reported in this review, only six compounds (**56**, **84**, **96**, **97**, **98**, and **99**) were tested in different coating matrices. Table 1 summarizes the AF potential of coatings containing these compounds and a comparison of the AF activity within the different coating formulations tested.

## 4. Discussion and Conclusions

Natural products, derived from marine and terrestrial sources, as well as several synthetic analogues have shown promising activity in the prevention of marine biofouling considering their activity against several macrofouling and microfouling species, including biofilm-forming species. Considering that these compounds should exert AF activity when incorporated into coatings, some research groups have been assessing the AF efficacy of the newly developed AF compounds into a coating matrix. 

Therefore, this review highlights the proof concept studies performed in the last five years (2019–2023) in different matrices, namely acrylic resins, rosin, polyurethane, silicone, and epoxy-based, laboratory-prepared, or commercial coatings, emphasizing AF performance.

Nearly 60 AF compounds were studied in acrylic-based coatings, followed by 27 compounds tested in rosin-based coatings. Natural polymers such as rosin have been emerging as sustainable alternatives. 

Most of the studies were conducted with “home-made” coatings. Few proof-of-concept studies were performed with real commercial coatings such as PU-based [18,21,76,79,84,85,86], foul-release PDMS [76,79,84,90], and epoxy-based coatings [93]. Commercial marine PDMS-based coating showed intrinsic anti-settlement activity against *M. galloprovincialis*, and no conclusive results could be drawn when compounds **96**–**98** were incorporated into these coatings [76,79]. The substitution of PDMS for RTV-PDMS overcame this issue as mussel larvae adhered to the free-compound RTV-PDMS coatings [76,79]. 

For the laboratory-prepared coatings, AF compounds were commonly mixed in powder with the other components. The commercial coatings used were all composed of two components, the base and the curing agent, and the AF compounds were commonly dissolved in organic solvents prior to the addition to the base of the coating. Then, the curing agent is added to the base containing the AF compound. Xylene was the most used solvent for the preparation of acrylic resin coatings, as well as the preparation of biodegradable polymer coatings, whereas for the preparation of rosin-based coatings, toluene was the solvent most reported. Considering the lipophilicity of these solvents, water-soluble AF compounds, although enjoying a non-toxic profile, may be challenging regarding formulation in polymeric coatings.

The bioactive compounds under study were added from 0.5% to 15%; the highest content of compounds was used in the acrylic resin-based coating and the lowest in rosin-based coatings. Chemical immobilization to the coatings was successfully applied to AF compounds **96** and **100** using MDI or TZA as crosslinkers [76,85]. The chemical immobilization of biocides was suggested as a strategy to provide a better long-term performance [94,95]. Also, with the same aim, nanotubes (HNTs) were used for compounds **103** and **104** [93].

Only a few compounds (**56**, **84**, **96**–**99**) were formulated in more than one polymeric matrix or had different formulations being tested for the same matrix (Table 1) [49,64,65,71,76,79,84,90]. These parallel studies are crucial to avoid the exclusion of a bioactive compound as an AF additive. For example, xanthone **98** was not compatible with PU-based coatings, probably due to its amine group, but this AF agent performed very well in RTV-PDMS, leading to 100% inhibition of the larvae settlement [79]. On the other hand, when the water soluble gallic acid derivative **96** was directly incorporated in PU, **96** failed in preventing the larvae settlement compared with the control, due to its fast release to seawater; however, when **96** was formulated with TZA, its release for seawater decreased and only 5% of the larvae were settled on the prepared PU coatings [76].

Coatings were commonly used to coat PVC and acrylic substrates. Most studies assessed the AF efficacy after submersion in the sea. The time of immersion of the AF coatings varied in the different studies, ranging from 1 month to 2 years. Nevertheless, some researchers studied the effectiveness of AF coatings using laboratory assays, namely bacterial assays [49,93] or in vivo *M. galloprovincialis* mussel larvae assays [18,21,76,79,84]. 

Few studies also assessed the release rate of bioactive compounds from the AF coatings, which is an important factor for the success of AF activity. She et al. evaluated the release rate of albofungin (**43**) from the coatings into artificial seawater for 35 days, showing that the release rate was generally low. Chiang et al. showed that the release of the synthetic derivative of butenolide (**86**) from the AF coating was much lower than natural butenolide (**84**), which can have impact on the AF potential. Vilas-Boas et al. showed that the incorporation of xanthone **97** into a PU-based coating resulted in a lower leaching than the incorporation of the same compound into a PDMS-based coating. In another study, Vilas-Boas et al. showed that the chemical immobilization of AF xanthone **100** into the PU-based coating resulted in zero leaching of this bioactive compound from the paint. Tonelli et al. also studied the release rate of compounds **103** and **104** from the epoxy coatings directly incorporated or loaded into HNTs and showed that the incorporation into HNTs prolonged the protective effect of these compounds against marine biofouling.

In conclusion, the studies highlighted in this review showed the potential application of natural and synthetic AF compounds in coatings, which, in most cases, displayed interesting AF performance in field assays. 

## Figures and Tables

**Table 1:** Summary of AF compounds tested in different coating matrices.

Compound	Coating Formulation	Antifouling Results	Main Differences between Tested Coatings
**56** (Capsaicin)	Acrylic-based resin with 1.5% and 3% of capsaicin [49].	After 48 h of exposure, coating containing 3% capsaicin had less colony-forming units of the gram-negative bacterium *Aeromonas salmonicida* than coating containing the same percentage of commercial biocide dichlofluanid.	Acrylic and HDPE coatings containing capsaicin showed effectiveness in inhibiting the growth of bacterial strains.
	HDPE coating with 1% and 2% of capsaicin [71].	After 48 h of exposure, HDPE coatings with 1% and 2% of capsaicin were able to decrease the growth of marine *Bacillus* sp. and coatings with 2% of capsaicin was also able to decrease the growth of *E. coli* bacterial strains by almost 100%. Uncoated plates or coated with HDPE free of capsaicin were not able to inhibit bacterial growth. After 1 week of exposure, HDPE coating with 2% of capsaicin was also able to reduce the adhesion of diatom *P. tricornutum*	
**84** (Butenolide)	Biodegradable synthetic polymer PLA-PU50 (composed of isophorone diisocyanate, poly(L-lactide) diol, 1,4-butanediol, dibutyltin dilaurate and THF) with 1%, 5%, 10%, and 20% of butenolide. Coatings combining the biodegradable polymer PLA-PU50 with binder rosin, with PLA-PU50:rosin ratios of 2:1, 1:1, and 1:2 with 10% of butenolide [64].	After three months of exposure, panels with 10% of butenolide and a PLA-PU50: rosin ratio and panels with 20% of butenolide without rosin showed similar performance, with a non-covered area of approximately 20%.	Coatings based on biodegradable synthetic polymers containing 10% of butenolide and combined with rosin or TSA showed higher AF effectivity than coatings based on biodegradable polymers with only butenolide. These results showed that the incorporation of rosin or TSA into the coatings increase the self-renewal rate of the polymer facilitating the long-term release of butenolide from the coating, thus increasing the AF efficiency.
	Biodegradable synthetic PU polymer (composed of PLA or PLGA as soft segments and different percentages of TSA (0%, 4%, 10, and 16%) as pendant groups) with 10% of butenolide [65].	After three months of exposure, PGLA coatings with 10% of butanolide and with higher content of TSA (16%) showed remarkable AF ability (with only approximately 7% of the panel area covered), whereas the other coatings with less than 16% of TSA were almost fouled (with approximately 95% of covered area).	
**96** (Gallic acid persulfate)	Acrylic coating with 0.5% and 1% of compound **96** added by direct incorporation or by chemical immobilization with TZA [76].	After 40 h of exposure to mussel *M. galloprovincialis* larvae, a larval settlement of 55% was observed when compound **96** was directly incorporated and only 15% when compound **96** was chemically immobilized.	Compound **96** was more compatible with PU coating than acrylic and RTV-PDMS coatings as it was possible to incorporate higher % of compound **96** in PU coatings [76]. However, the lowest release values (0.35%) [90] and the best anti-settlement activity were obtained in RTV-PDMS. The chemical immobilization of compound **96** decreased the release of compound **96** for seawater (two times lower in PDMS coatings, five times lower in PU coatings), compared with direct incorporation [76]. The chemical immobilization of compound **96** with TZA, either in acrylic, PU, and RTV-PDMS coatings significantly decreased the larval settlement compared with direct incorporation [76], with no settlement (100% inhibition) observed on RTV-PDMS.
	PU coating with 2% of compound **96** added by direct incorporation or by chemical immobilization with TZA [76].	After 40 h of exposure to mussel *M. galloprovincialis* larvae, a decrease in larval settlement was only observed when compound **96** was chemically immobilized in PU coating	
	PDMS and RTV-PDMS coating with 0.56% of compound **96** added by direct incorporation or by chemical immobilization with TZA [76,90].	Compound-free PDMS coating had intrinsic anti-settlement activity, therefore no conclusive results were possible to be made when compound **96** was added. After 40 h of exposure to larvae of mussel *M. galloprovincialis*, RTV-PDMS coatings with compound **96** chemically immobilized had no larvae settled on.	
**97** (Xanthone)	Acrylic coating with 0.55% of compound **97** [79].	After 40 h of exposure to mussel *M. galloprovincialis* larvae, 30% of mussel larval settlement was observed for coating with compound **97**, whereas compound-free coating showed 50% of larval settlement.	PU-based coatings allowed the incorporation of higher % of compound **97.** Despite the lower % of compound **97** in RTV-PDMS, the best anti-settlement activity was obtained in RTV-PDMS. PU-based coating resulted in a lower release of compound **97** (1.67%) than PDMS-based coating (16.7%). Overall, PU seems the most suitable matrix for compound **97**.
	PU coating with 2% of compound **97** [79].	After 40 h of exposure to mussel *M. galloprovincialis* larvae, only 10% of mussel larval settlement was observed, whereas compound-free coating showed 35% of larval settlement.	
	PDMS and RTV-PDMS coatings with 0.52–0.53% of compound **97** [79].	Compound-free PDMS coating had intrinsic anti-settlement activity, therefore no conclusive results were possible to be made when compound **97** was present. After 40 h of exposure to mussel *M. galloprovincialis* larvae, no mussel larval settlement (0%) was observed for RTV-PDMS coating containing compound **97**, in contrast to the compound-free RTV-PDMS coatings which exhibited 10% of larval settlement.	
**98** (Xanthone)	Acrylic coating with 1% of compound **98** [79].	After 40 h of exposure to mussel *M. galloprovincialis* larvae, 20% of mussel larval settlement was observed on coatings containing compound **98**, in contrast to the compound-free coating in which a larval settlement of 50% was observed.	PDMS-based coating resulted in high release of compound **98** (25%). These results indicate a possible short time effect of this compound in the PDMS coating. Compound **98** was more compatible with acrylic coatings than PDMS and RTV-PDMS coatings as it was possible to incorporate compound **98** in higher % in acrylic coatings than in PDMS and RTV-PDMS coatings. Even though the lower % of compound **98** in RTV-PDMS, the best anti-settlement activity was obtained in RTV-PDMS. Compound **98** was not compatible with PU coatings
	PDMS and RTV-PDMS coatings with 0.53–0.55% of compound **98** [79].	For PDMS coating, no settlement was observed for compound-free coating as well as coatings containing compound **98**. After 40 h of exposure to mussel *M. galloprovincialis* larvae, no mussel larval settlement (0%) was observed for RTV-PDMS coating containing compound **98**, whereas the compound-free RTV-PDMS coatings displayed 10% of larvae settlement.	
**99** (Methyl deoxycholate)	PU coating with 0.58% of compound **99** [84].	After 40 h of exposure to mussel *M. galloprovincialis* larvae, PU coating containing compound **99** was more effective than compound-free coating and coating with the biocide Econea®.	Compound **99** showed good and similar compatibility with both PU and PDMS coatings at contents up to 0.58 wt.%. PU and RTV-PDMS coatings containing compound **99** were more effective against the settlement of the *M. galloprovincialis* mussel larvae than compound-free coating.
	RTV-PDMS coating with 0.58% of compound **99** [84].	After 40 h of exposure to mussel *M. galloprovincialis* larvae, no mussel larval settlement was observed on RTV-PDMS coatings containing Econea® or compound **99**, whereas some attachment was found for compound-free coating.	

## Data Availability

Not applicable.
